# War-related and minority-related stressors and posttraumatic stress symptoms among Palestinian Arab grandmothers in Israel: a resource-based mediation model

**DOI:** 10.3389/fpsyt.2026.1833476

**Published:** 2026-07-21

**Authors:** Hanan AboJabel, Michal Levinsky, Malitta Engstrom, Miriam Schiff

**Affiliations:** 1 Paul Baerwald School of Social Work and Social Welfare, Hebrew University of Jerusalem, Jerusalem, Israel; 2 School of Social Policy & Practice, University of Pennsylvania, Philadelphia, PA, United States

**Keywords:** Arab grandmothers, armed conflict, caregiving, ethnic minority, minority-related stress, posttraumatic stress symptoms, resource gain, resource loss

## Abstract

**Background:**

Exposure to armed conflict is associated with elevated posttraumatic stress symptoms (PTSS). Ethnic minority groups may also face minority-related stressors, such as discrimination and silencing, that compound psychological vulnerability. Conservation of resources theory suggests that loss of valued personal and social resources may help explain PTSS under prolonged adversity. Palestinian Arab grandmothers in Israel remain understudied despite central roles in multigenerational collectivist family systems, which may intensify during collective threat and heighten vulnerability to posttraumatic stress.

**Objective:**

This study examined associations of grandchild caregiving, war-related trauma, and minority-related stressors with PTSS among Palestinian Arab grandmothers in Israel, and whether resource loss and gain mediated these associations.

**Methods:**

A cross-sectional study was conducted among 112 Palestinian Arab grandmothers (Mean age = 58.50, SD = 6.60) during a period of continuous war-related traumatic stress. Data were collected using a structured online questionnaire.

**Results:**

Mean PTSS was 2.27 (SD = 1.41, range=0–4), indicating a moderate PTSS level. Most participants (93.7%) reported at least one war-related traumatic event, and 34.8% reported at least one minority-related stressor. Weekly grandchild caregiving hours were not associated with PTSS. In regression analyses, minority-related stress remained significantly associated with PTSS (β = .20, p<.05), whereas war-related exposure did not (β = .16, p >.05). Resource loss remained significant in the final model (β=.25, p<.05), which explained 22% of PTSS variance (R^2^=.22, p <.001). Mediation analysis showed a significant indirect effect of minority-related stress on PTSS through resource loss (ab=.05, 95% CI [0.0012,0.1196]).

**Conclusions:**

Interventions targeting minority-related stress and preserving psychosocial resources may benefit Palestinian Arab grandmothers in contexts of ongoing conflict.

## Introduction

Trauma exposure in contexts of armed conflict and sociopolitical instability is consistently linked to poorer psychological outcomes, including elevated posttraumatic stress symptoms (PTSS) among civilians (1, 2). Similar patterns have also been documented among older adults (3, 4). Yet one subgroup remains comparatively underexamined: grandmothers. In many societies, particularly traditional and collectivistic ones, grandmothers occupy pivotal positions within multigenerational family systems through their active involvement in grandchild caregiving and childrearing (5, 6). During times of war or armed conflict, grandparenthood may intensify, as grandparents increase their emotional and practical involvement to foster stability, a sense of safety, and resilience for grandchildren through routine adjustments, greater home-based caregiving, and reassurance during siren-related stressors (7). Such intensified caregiving may increase grandmothers’ vulnerability to adverse mental health outcomes, as greater caregiving intensity and burden have been linked to increased psychological distress among caregiving grandparents (8).

Nevertheless, grandmotherhood under prolonged conditions of war and armed conflict has received only limited scholarly attention (7, 9–11).This gap is especially salient among grandmothers from ethnic minority groups, who may face compounded stressors associated with both prolonged armed conflict and minority status (12). To the best of our knowledge, PTSS responses in this population have not yet been examined, leaving an important gap in understanding their psychological vulnerability in contexts of ongoing sociopolitical instability.

The present study focuses on Palestinian Arab grandmothers in Israel in the period following the October 7 events and the subsequent war. This population is embedded in collectivistic social structures in which intergenerational ties and family caregiving are highly valued, and grandmothers often occupy a central supportive role within extended family networks (13). Furthermore, Palestinian Arab grandmothers are situated at the intersection of multiple and cumulative stress exposures. As members of a minority group, they are often exposed to structural stressors, including socioeconomic disadvantage, persistent health disparities, and ongoing experiences of discrimination and social exclusion (14–16; 17, 18).

In the period following October 7, these chronic minority status stressors were amplified and compounded by war-related pressures. At a broader societal level, the war had far-reaching effects across Israeli society, undermining personal security, social cohesion, and trust in state institutions and contributing to a sustained sense of instability (19, 20). In addition, research conducted in the aftermath of the October 7 attack indicates increased psychological distress in Israel following exposure to traumatic events related to the attack itself, such as being present in affected areas and experiencing the injury, kidnapping, or death of a loved one. Higher prevalences of PTSD, depression, and anxiety were reported among Arab citizens compared to Jewish citizens (21). Furthermore, a study conducted among residents of southern Israel living under chronic missile threat, characterized by repeated rocket fire, warning sirens, and the need to seek shelter, found higher levels of PTSD among Arab Bedouins compared to Jewish residents (22).

Beyond direct exposure, Arabs in Israel also faced distinct stressors related to minority status. These experiences included processes of silencing during the war period, such as summons for questioning and arrests following social media posts expressing sorrow over events in Gaza that were framed as incitement (23). In this context, AboJabel and Ayalon (24) found that older Arab adults refrained from publicly expressing emotions or viewpoints related to the war due to deep fears of personal consequences, such as interrogation or arrest, as well as familial consequences, including concerns about harm to their children through employment-related repercussions such as workplace retaliation or dismissal.

To understand how exposure to war-related traumatic events, alongside stressors associated with minority status, is linked to PTSS among Palestinian Arab grandmothers in Israel, the present study draws on the stress process model, which conceptualizes psychological outcomes as the result of the interplay between background and contextual conditions, stressors, and psychosocial resources (25). In the current study, background and contextual factors include sociodemographic characteristics as well as aspects of the grandparenting context, such as socioeconomic resources and the extent of caregiving involvement with grandchildren (7, 8); stressors include war-related traumatic events that are shared with the broader population (26), as well as minority-related stressors that are more specific to Arabs in Israel (16, 24); the primary outcome dependent variable is PTSS (21).

Importantly, the model highlights psychosocial resources as central mediating mechanisms that help explain why some individuals develop higher levels of symptoms than others. Accordingly, the study focuses on resource loss and resource gain as potential pathways linking stress exposure to PTSS. In this context, conservation of resources theory (27) posits that psychological stress arises when individuals experience an actual or threatened loss of valued resources, or when they fail to obtain resources following significant investment. Such resources may include a sense of control and self-worth, supportive social relationships, and life conditions perceived as meaningful and sustaining. Accordingly, resource loss is expected to increase vulnerability to PTSS, whereas resource gain may reduce the impact of stressors even under conditions of prolonged adversity (25, 27).

Based on this theoretical framework and existing research, the present study had two main objectives. First, we examined whether war-related traumatic exposure and minority-related stressors were associated with posttraumatic stress symptoms (PTSS) among Palestinian Arab grandmothers in Israel, while accounting for their unique caregiving role within multigenerational family systems. Grandchild caregiving involvement was operationalized using weekly hours of care, dichotomized at 5 hours per week (<5 vs. ≥5), consistent with prior literature defining regular grandchild care as at least 5 hours per week (28). Second, we tested whether resource loss and resource gain mediated these associations. We hypothesized that greater caregiving involvement (≥5 weekly hours) would be associated with higher PTSS (H1). We further hypothesized that greater exposure to war-related traumatic events and minority-related stressors would be associated with higher PTSS above and beyond background and contextual factors (H2). Finally, we hypothesized that these associations would be mediated by higher resource loss and lower resource gain (H3).

## Methods

### Design and procedure

This cross-sectional study used a convenience sample of Palestinian Arab grandmothers in Israel and was conducted through an anonymous online questionnaire. Participants were eligible if they were grandmothers aged 45 years or older. No additional exclusion criteria were applied beyond failure to meet this criterion. A total of 137 individuals responded to the questionnaire. Of this group, 112 participants were included in the final analytic sample; 25 responses were excluded because the questionnaire was incomplete or because participants did not meet the eligibility criteria. The sample size was estimated using G*Power (version 3.1) for a multiple linear regression model with five predictors, corresponding to the central predictors specified in the study’s conceptual model. Assuming a medium effect size (f^2^ = 0.15), an alpha level of.05, and statistical power of.80, the required sample size was 92 participants. The final sample of 112 participants exceeded this requirement. This sample size also meets Green’s (29) recommendations for multiple regression, namely N ≥ 50 + 8m for testing the overall model fit (50 + 8 × 5 = 90) and N ≥ 104 + m for testing individual regression coefficients (104 + 5 = 109), supporting the adequacy of the sample size for the planned exploratory multivariable analyses.

Recruitment relied on a mixed approach. Twenty-one participants were recruited through the survey company iPanel, which facilitated access to eligible respondents. Additional recruitment was conducted through social media networks, including Facebook and WhatsApp. The use of a convenience sample and multiple recruitment channels was motivated by practical challenges in recruiting women aged 50 and older for survey research, including lower levels of formal education, limited digital literacy, and heightened mistrust toward state institutions and formal systems, including researchers, which may reduce willingness to participate in surveys (14).

### Measures

*Contextual variables* included religion (Muslim, Christian, or Druze), age, education (less than high school, high school, or more than high school), financial status (financially struggling vs. financially managing well), marital status (married vs. not married), and place of residence (north, south, center, or Jerusalem). In addition, participants reported their self-rated health using a single-item measure (30) rated from 1 (very good) to 5 (very poor). The grandparenting context included the number of grandchildren and weekly hours of grandchild care (<5 vs. ≥5 hours per week).

### Stressors

*War-related traumatic exposure* was assessed using a 16-item yes/no checklist adapted and expanded from Shrira & Palgi (26). The checklist was revised to reflect the prolonged nature of the conflict and to capture additional context-specific stressors (e.g., property damage, disruption to livelihood, hostage-related events involving family or friends, and participation in protests calling for the return of hostages). Participants indicated whether they had experienced each event, and endorsed items were summed to create a total war-related traumatic exposure score (range: 0 to 16), with higher scores indicating greater exposure.

*Minority-related wartime stressors* were assessed using a two-item yes/no checklist capturing experiences since the October 7 events. Items assessed whether the participant or someone close to her (family, friends, or acquaintances) had been arrested or questioned due to expressing an opinion about the war, and whether the participant or someone close to her had experienced discrimination or hostile treatment, including harm to employment conditions or job termination. Endorsed items were summed to create a total minority-related stress exposure score (range: 0 to 2), with higher scores indicating greater exposure.

### Mediators

*Resource loss* was assessed using seven items derived from Hobfoll’s Conservation of Resources (COR) framework (27, 31). The items covered key domains relevant to the ongoing war (e.g., trust in the government, meaning in life, social support, self-worth, perceived control, hope for the future, and coping ability). Participants rated the extent of loss in each domain on a 4-point scale from 1 (no loss) to 4 (very great loss). Responses were summed to create a total score (range: 7–28), with higher scores indicating greater resource loss. In the present study, the scale demonstrated good internal consistency (α = .85).

*Resource gain* was assessed using the same seven COR-based items (27, 31). Participants rated the extent to which each resource had been strengthened over the past year on a 4-point scale from 1 (not strengthened at all) to 4 (greatly strengthened). Responses were summed to create a total score (range: 7–28), with higher scores indicating greater resource gain. In the present study, the scale demonstrated good internal consistency (α = .86).

### Dependent variable

*PTSS* were assessed using the Primary Care PTSD Screen (PC-PTSD; 32), a brief screening instrument consisting of four items reflecting core PTSS. Participants responded “yes” or “no” to each item, and a total score was calculated by summing endorsed items (range: 0–4), with higher scores indicating greater PTSS. In the present study, the scale demonstrated good internal consistency (KR-20 = .71; α = .71).

### Data analysis

Data analyses were conducted using IBM SPSS Statistics (version 31). Data were screened prior to analysis, and missingness was low. Therefore, no imputation was applied, and analyses were conducted with available data. Descriptive statistics were performed to describe the sample and key study variables, including means, standard deviations, ranges, and percentages. Bivariate associations with PTSS were examined using Pearson correlation coefficients for continuous variables and independent-samples t tests or 1-way analyses of variance (ANOVA) for categorical variables, as appropriate. Effect sizes for group comparisons were reported using Cohen’s d for independent-samples t tests and eta-squared (η^2^) for one-way ANOVAs. To examine potential multicollinearity, variance inflation factors (VIFs) were calculated. Values greater than 5 were considered problematic (33), and in the current study, VIF values ranged from 1.06 to 1.24, indicating no multicollinearity concerns. Hierarchical multiple regression analyses were then conducted to examine factors associated with PTSS; only variables that showed statistically significant bivariate associations with PTSS were entered into the regression models. Variables were entered in blocks, beginning with the contextual covariate, followed by the stress exposure variables, and finally the resource variables (resource loss and resource gain). Standardized regression coefficients (β) and explained variance (R^2^) were reported. Mediation analyses were conducted using Hayes’ PROCESS macro in SPSS (Model 4). Standardized coefficients were reported for all paths. Indirect effects were estimated using 5,000 bootstrap resamples with 95% bias-corrected confidence intervals, and statistical significance was determined when the confidence interval did not include zero (34).

## Results

### Sample characteristics

Descriptive characteristics of the study sample are presented in **Table 1**. The sample consisted of 112 grandparents, the majority of whom identified as Muslim (88.3%), followed by Christian (9.0%), and Druze (2.7%). Participants’ mean age was 58.50 years (SD = 6.60, range = 45 to 86). Regarding education, 33.6% of participants reported less than a high school education and 66.4% of participants reported high school or more. Most participants reported experiencing financial difficulties (85.3%), and 77.3% of participants were married. Participants resided across several regions in Israel, with the largest proportion living in Jerusalem (54.1%), followed by the North (36.7%), the Center (Central District; 4.6%), and the South (4.6%). Participants reported a moderate level of self-rated health (M = 2.98, SD = 0.91, range = 1 to 5), with higher scores indicating poorer perceived health. In terms of the grandparenting context, participants reported an average of 6.58 grandchildren (SD = 6.43, range = 1 to 35), and weekly hours of grandchild care were categorized into less than 5 hours (27.1%) and 5 hours or more (72.9%). Regarding stressors, the vast majority of participants (93.7%) reported exposure to at least one war-related traumatic event (Mean = 1.55, SD = 0.95, range = 0 to 5), whereas 34.8% reported exposure to at least one minority-related event (Mean = 0.49, SD = .73, range = 0 to 2). Finally, participants reported moderate levels of both resource loss (Mean = 14.27, SD = 5.23 range = 7–28) and resource gain (M = 14.03, SD = 4.72, range = 7–28), as well as moderate levels of PTSS (Mean = 2.27, SD = 1.41, range = 0–4).

**Table 1:** Sample characteristics and bivariate associations with PTSS (N = 112).

Mean (SD, range)/ %	Bivariate Tests (r / t / F)	η^2^/ Cohen’s	
**Contextual variable**			
Religion %			
Muslim	88.3	2.27 (1.39)^NS^	.02
Christian	9.0	2.40 (1.57)	
Druze	2.7	1.00 (1.00)	
Mean *age* (SD, Range)	58.50 (6.60, 45.00-86.00)	-.10^NS^	
Education %			
Less than High School	33.6	2.32 (1.41)^NS^	1.41
High School and More	66.4	2.29 (1.40)	
Financial Status %			
Financially Struggling	85.3	2.44 (1.39)^NS^	1.36
Financially Managing Well	14.7	1.60 (1.18)	
Marital Status (%)			
Married	77.3	2.24 (1.40)^NS^	1.41
Not Married	22.7	2.33 (1.46)	
Place of Residence (%)			
North	36.7	2.17 (1.37)^NS^	0.2
South	4.6	1.60 (2.19)	
Center	4.6	2.40 (1.81)	
Jerusalem	54.1	2.48 (1.30)	
Mean *Self-Rated Health* (SD, Range)	2.98 (0.91, 1.00-5.00)	.22*	
Mean *Number of Grandchildren* (SD, Range)	6.58 (6.43, 1.00-35.00)	-.05^NS^	
*Hours of Grandchild Care* (%)			
<5	27.1	1.93 (1.53)^NS^	1.41
=>5	72.9	2.35 (1.36)	
Stressors			
Mean *War-Related Trauma Exposure* (SD, Range)	1.55 (.95, .00-5.00)	.21*	
Mean *Minority-Related Stress Exposure* (SD, Range)	.49 (.73, .00-.2.00)	.22*	
Resources			
Mean *Resource Loss* (SD, Range)	14.27 (5.23, 4.00-28.00)	.37**	
Mean *Resource Gain* (SD, Range)	14.03 (4.72, 7.00-28.00)	-.20*	
Dependent Variable			
Mean *PTSS* (SD, Range)	2.27 (1.41, .00-4.00)	–	

Pearson’s r was used for age, self-rated health, number of grandchildren, war-related trauma exposure, minority-related stress exposure, resource loss, and resource gain. Independent-samples t tests were used for education, financial status, marital status, and weekly hours of grandchild care. One-way ANOVAs were used for religion and place of residence. Cohen’s d was reported for t tests, and η^2^ was reported for ANOVAs. **p<.001, *p<.05, NS: not significant.

### Bivariate associations with PTSS

Bivariate analyses indicated several statistically significant associations with PTSS. Poorer self-rated health was associated with higher PTSS levels (r = .22, p <.05). In addition, greater war-related trauma exposure and greater minority-related stress exposure were each linked to higher PTSS (r = .21 and r = .22, respectively; p <.05). Resource loss was positively associated with PTSS (r = .37, p <.001), whereas resource gain was negatively associated with PTSS (r = −.20, p <.05). Regarding the other variables examined, none were significantly associated with PTSS (p >.05). Specifically, weekly grandchild care hours were not significantly related to PTSS: participants providing fewer than 5 hours of care reported a mean PTSS of 1.93 (SD = 1.53), compared to 2.35 (SD = 1.36) among participants providing 5 hours or more (P >.05). Accordingly, H1 was not supported.

### Hierarchical multiple regression analyses of factors associated with PTSS

In hierarchical multiple regression analyses (**Table 2**), Step 1 indicated that poorer self-rated health was significantly associated with higher PTSS (β = .22, P <.05), and this model explained 5% of the variance in PTSS (R^2^ = .05, P <.05). In Step 2, self-rated health (β = .29, P <.05) and minority-related trauma events (β = .20, P <.05) were significantly associated with PTSS, whereas war-related trauma events were not (β = .16, P >.05); this step explained 13% of the variance (R^2^ = .13, P <.001). Accordingly, H2 was partially supported. In Step 3, resource loss was significantly associated with higher PTSS (β = .25, P <.05), while self-rated health, war-related trauma exposure, minority-related stress exposure, and resource gain were not statistically significant (P >.05); the final model explained 22% of the variance in PTSS (R^2^ = .22, P <.001).

**Table 2:** Hierarchical multiple regression analysis explaining variance in PTSS (N = 112).

Step 1.	
Self-rated health	0.22*
R^2^	.05*
Step 2.	
Self-rated health	.29*
War-related trauma events	.16
Minority-related trauma events	.20*
R^2^	.13**
Step 3.	
Self-rated health	.14
War-related trauma exposure	.11
Minority-related stress exposure	.17
Resource loss	.25*
Resource gain	-.15
R^2^	.22**

NS: not significant, *p < 0.05, **p < 0.01.

Accordingly, the mediation model was estimated using PROCESS Model 4, with minority-related stress exposure as the independent variable, resource loss as the mediator, PTSS as the dependent variable, and self-rated health as the covariate. War-related trauma exposure and resource gain were not included because they were not significantly associated with PTSS in the multivariable model.

### Mediation of the association between minority-related stress and PTSS

**Figure 1:**
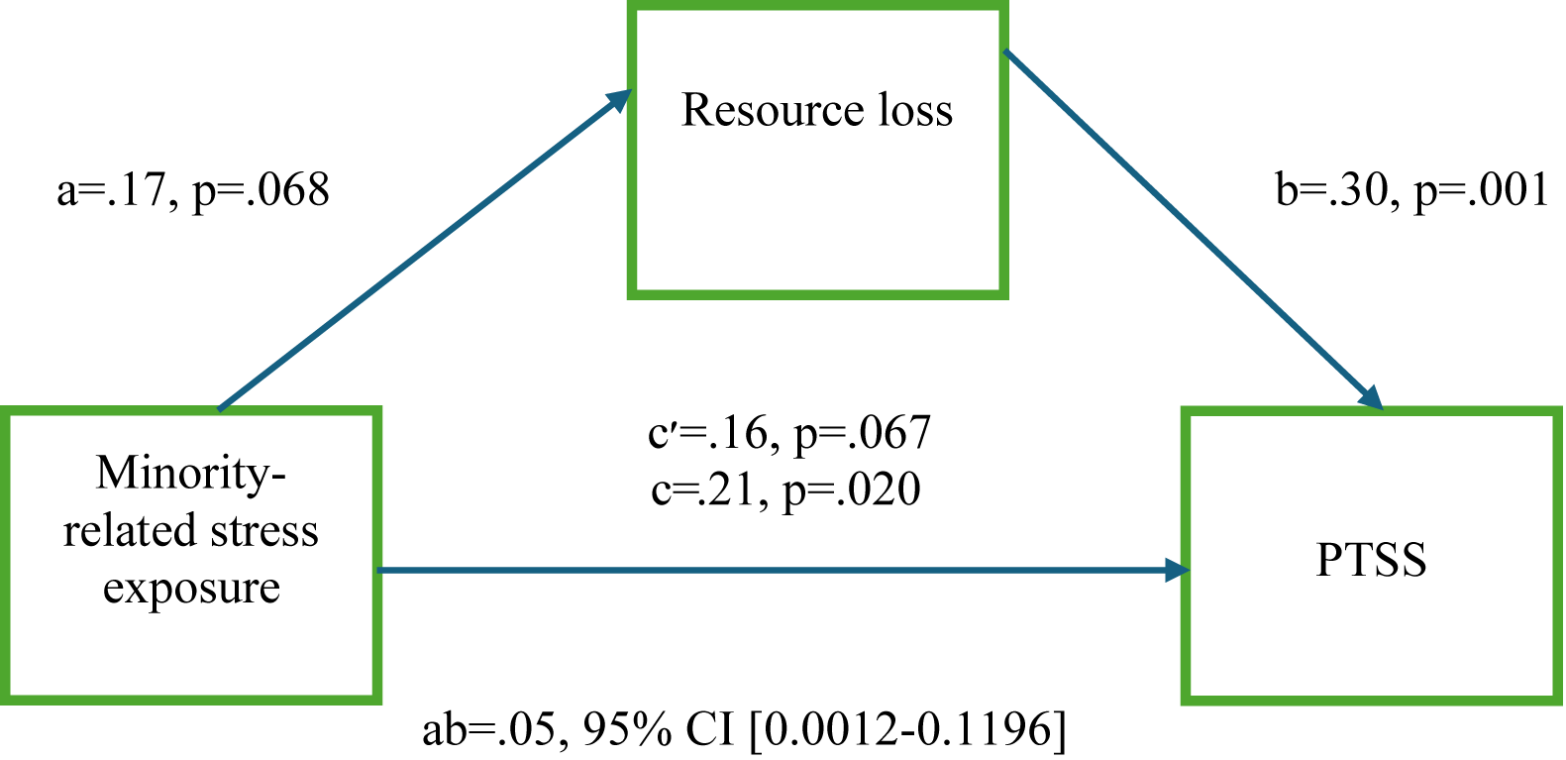
Mediation model of resource loss between minority-related stress exposure and PTSS. The model controls for self-rated health. All displayed path coefficients (a, b, c, and c′) are standardized coefficients (b).

Results of the mediation analysis are presented in **Figure 1**. Minority-related stress exposure was marginally associated with resource loss (path a: β= .17, P = .068), and resource loss was significantly associated with higher PTSS (path b: β= .30, P = .001). The total effect of minority-related stress exposure on PTSD symptoms was significant (path c: β= .21, P = .020). When resource loss was included in the model, the direct effect was reduced and became marginal (path c′: β= .16, P = .067). Bootstrapping indicated a significant indirect effect through resource loss (ab = .05, 95% CI [.0012,.1196]), supporting partial mediation. Accordingly, H3 was partially supported.

## Discussion

The current study examined associations between grandchild caregiving involvement, war-related traumatic exposure, minority-related stressors, and PTSS among Palestinian Arab grandmothers in Israel, as well as the mediating role of resource loss and gain. Several important findings emerged. First, weekly hours of grandchild care (≥5 hours versus <5 hours) were not significantly associated with PTSS. One possible explanation for this finding relates to the cultural context of the study population. In Palestinian Arab society, active involvement in caring for grandchildren is often perceived as a normative and expected component of the grandmother’s role within the extended family (13). Accordingly, the frequency of caregiving may not necessarily reflect psychological burden, but rather culturally embedded familial responsibility. Future research should examine not only the extent of caregiving, but also its qualitative dimensions, such as perceived burden and the subjective meaning attributed to caregiving, which may be more closely associated with psychological outcomes.

Second, both war-related traumatic exposure and minority-related stressors were associated with PTSS at the bivariate level, consistent with prior research documenting elevated posttraumatic stress among civilians exposed to armed conflict and sociopolitical violence (1, 2, 21). The present results extend the literature by focusing specifically on Palestinian Arab grandmothers, a population that has received limited empirical attention despite their central familial role within collectivist social structures. However, in Step 2 of the regression analyses, after controlling for self-rated health, only exposure to minority status-related stressors remained significantly associated with PTSS, exposure to war-related traumatic events was no longer significant. This pattern suggests that beyond shared exposure to war events, stressors unique to minority status, such as fear of arrest, silencing, and discrimination, may constitute a particularly substantial psychological burden for Palestinian Arab grandmothers. This finding is consistent with research among other ethnic minority groups showing that discrimination is associated with elevated PTSS and poorer mental health (35, 36) and aligns with the stress process model, which highlights the impact of chronic strains on mental health (25, 37).

Third, at the bivariate level, both resource loss and resource gain were significantly associated with PTSS, such that greater perceived loss was linked to higher PTSS and greater resource gain was linked to lower PTSS. However, in the hierarchical regression model, only resource loss remained significantly associated with PTSS. Higher perceived loss of valued psychosocial resources was associated with higher levels of PTSS. This finding is consistent with conservation of resources theory (27), which posits that stress reactions intensify when individuals experience actual or threatened loss of valued resources. Resource gain did not remain statistically significant in the multivariable model, suggesting that in the context of ongoing conflict and sociopolitical strain, resource depletion may be more psychologically consequential than resource strengthening. This interpretation aligns with prior findings indicating that resource loss exerts a stronger impact on psychological distress than resource gain. For example, Egozi Farkash et al. (38) found that resource loss significantly predicted COVID-19-related traumatic symptoms, whereas resource gain did not remain significant in the multivariable model, concluding that loss tends to be more impactful than gain in times of crisis.

Importantly, mediation analyses indicated that resource loss partially mediated the association between minority status–related stress exposure and PTSS. Taken together, these results support the interpretation that minority-related stress contributes to PTSS in part through its impact on the perceived erosion of psychosocial resources; experiences of discrimination, silencing, and minority-based stress may undermine core psychological resources and thereby increase vulnerability to PTSS. Similar findings were reported in a study conducted in Israel among Bedouin members of the Israel Defense Forces, which found that personal resource loss mediated the association between trauma exposure and PTSD, depression, and anxiety symptoms (39).

The study concludes that among Palestinian Arab grandmothers in Israel, it is not only exposure to war-related traumatic events that is central to understanding PTSS, but also minority status-related stressors and the way they are intertwined with the erosion of psychosocial resources. A key finding is that resource loss, including diminished trust, meaning, perceived control, and hope, was associated with PTSS and partially mediated the relationship between minority-related stress and these symptoms. This pattern suggests a pathway through which minority-related stress may undermine internal and social resources, thereby increasing vulnerability to PTSS. The study makes two main contributions: first, it addresses a research gap by focusing on an understudied population with central, but empirically overlooked familial roles, particularly in the context of political trauma; and second, it identifies a theoretically grounded and clinically relevant mechanism for reducing PTSS: the importance of preserving and restoring psychosocial resources as a key target for intervention among grandmothers living under ongoing conflict and minority-related stress.

Alongside its contributions, the study has several limitations that should be considered when interpreting the findings. First, the cross-sectional design limits the ability to draw causal conclusions or determine the temporal ordering between stress exposure, resource loss, and PTSS. Second, the sample size (N = 112), together with the use of a convenience sample and online recruitment, may affect the generalizability of the results, particularly to older women with limited digital access or lower willingness to participate in online survey research. In addition, the sample included a relatively large proportion of participants residing in Jerusalem; regional variation within the Palestinian Arab population in Israel may not be fully reflected in the present findings. Third, although the bootstrapped indirect effect through resource loss was statistically significant, the association between minority-related stress exposure and resource loss reached only marginal significance. Therefore, this mediation pathway should be interpreted with caution. Fourth, PTSS was measured using the four-item PC-PTSD, a brief screening tool. Although its brief format was appropriate for older participants completing an anonymous online wartime survey, it was not used to establish a clinical diagnosis. Rather, its summed score was treated as an ordinal indicator of PTSS symptom burden, consistent with prior quantitative studies (e.g., 40, 41). Future research should include larger and more geographically diverse samples, more accessible data collection methods such as interviewer-administered questionnaires conducted in person or by telephone, and more comprehensive dimensional measures of PTSS. Despite these limitations, this study contributes to understanding the multidimensional stressors experienced by Palestinian Arab grandmothers in Israel, the factors associated with PTSS, and the importance of preserving psychosocial resources in interventions addressing war- and minority-status-related stress in this population.

## Data Availability

The raw data supporting the conclusions of this article will be made available by the authors, without undue reservation.
